# Ten Myths About the Effect of Social Media Use on Well-Being

**DOI:** 10.2196/59585

**Published:** 2024-11-25

**Authors:** Jeffrey A Hall

**Affiliations:** 1 Department of Communication Studies University of Kansas Lawrence, KS United States

**Keywords:** social media, well-being, health promotion, depressive disorder, depression, anxiety, adolescent, mental health

## Abstract

This viewpoint reviews the empirical evidence regarding the association between social media use and well-being, including life satisfaction and affective well-being, and the association between social media use and ill-being, including loneliness, anxiety, and depressive symptomology. To frame this discussion, this viewpoint will present 10 widely believed myths about social media, each drawn from popular discourse on the topic. In rebuttal, this viewpoint will offer a warranted claim supported by the research. The goal is to bring popular beliefs into dialogue with state-of-the-art quantitative social scientific evidence. It is the intention of this viewpoint to provide a more accurate and nuanced claim to challenge each myth. This viewpoint will bring attention to the importance of using rigorous scientific evidence to inform public debates about social media use and well-being, especially among adolescents and young adults.

Claims about the harms of social media are everywhere. Many are taken as fact. In the face of this certainty, it is useful to remember that it was once taken for granted that serial dramas on the radio, comic books, going to the cinema, and arcade games were all once considered to be undeniably harmful, particularly for the youth [1]. This is not to discount the legitimate interests of the public and policy makers to understand whether and how social media influence well-being. Locating the latest and most accurate research information is challenging. Evidence on the topic is endlessly updated, reviewed, and debated by researchers from fields ranging from psychology and communication through education and computer science to neuroscience and economics.

To understand this new form of an old controversy and to summarize complex and scattered academic evidence, this paper will use a myth versus warranted claim structure. This paper will present 10 myths about the harms of social media. Each myth is directly quoted or paraphrased from public discourse, including op-eds, podcasts, claims of politicians, and news headlines. In response to each myth, a warranted claim supported by peer-reviewed research will be offered. Meta-analyses, studies with large or representative samples, and studies using longitudinal methods will be prioritized as sources for supporting and justifying each warranted claim.

Social media are platforms that enable searchable and publicly distributed content, including text, photos, memes, and videos [2]. Social media are often identified by a branded platform name (eg, Instagram). Users create content and this content is transmitted in a decentralized fashion. Users—to varying degrees depending on the platform—decide what is shared and reshared. Platforms vary considerably as to whether it is normative for users to share content, or whether the content is typically distributed by prominent accounts or users (eg, influencers or content creators). Meta-analyses suggest that social network sites, particularly Facebook, are the most studied social media [2-4]. Thus, most meta-analytic claims are drawn from evidence from decades of research on Facebook use, primarily among American college students [2-4]. Social media platforms that distribute video content (eg, TikTok or YouTube) have recently become quite popular [5], but are less well-represented in the research on well-being. Whenever possible, this viewpoint will focus on time using social media, rather than screen time generally, which often includes TV, gaming, and internet use.

In this paper, well-being will be defined as including both eudaemonic and hedonic well-being. While the former focuses on meaning, connection, and life purpose, the latter focuses on pleasure, enjoyment, and entertainment. Both well-being and ill-being will be examined. Well-being is typically measured in social media research as life satisfaction, positive emotions, and the absence of negative emotions, while ill-being is typically measured as the presence of mental health symptomology (eg, anxiety, depression, or loneliness) [4,6].

This paper will focus on the effects of social media on the well-being of users as an aggregate. Social science research reports effect sizes, typically expressed as a correlation or mean difference. By nature, the goal of quantitative social science is to document the association between 2 (or more) variables, not to document the singular experience of each participant in the study. Thus, trends in the data, reported as aggregate effects, are not certainties that equally apply to all people. No association between 2 variables is equally true for every single person in the sample. Thus, it can be true that the warranted claims are accurate for the whole and that they do not perfectly align with every individual’s experience.

Furthermore, this paper will not examine cases of real-world harms conducted through or enabled by social media. Social media are used to facilitate the sale of illegal substances, to coordinate sex trafficking, to plan terrorism, and to send death threats. Each activity is illegal whether conducted through a mobile device or social media platform or on landline telephone or mailed post. When this paper speaks about the effects of social media, it will focus on its typical and mundane uses (ie, how most people use social media most of the time), not social media when used as a telecommunications channel to commit illegal acts. If the reader’s concerns about social media are focused on illegal activities, this paper will not be informative. If the reader feels that the harms of social media when used to commit illegal acts outweigh the influence of mundane and typical uses of social media on users’ well-being, then this paper will not be persuasive.

- *Myth one: There is undeniable evidence that time spent on social media has a toxic effect on its users.*
- *Warranted claim one: Time spent on social media does not have a strong effect on the well-being of its users.*

There are 2 decades of research on the association between social media and well-being. A comprehensive meta-analysis that reviewed 226 studies published between 2006 and 2018, including 275,728 participants and 1279 effect sizes, reported a weighted mean effect size of *r*=.01, which is no different from 0 [4]. An analysis of 3 data sets, including 355,000 adolescents, found that the association between social media use and well-being accounts for, at most, 0.4% of the variance in well-being, which the authors conclude is of “little practical value” [7]. Another large study of adolescent users concluded that the association was “too small to merit substantial scientific discussion” [8]. A longitudinal study that measured social media use through an app installed on participants’ mobile devices found no associations between any measures of Facebook use and loneliness or depression over time [9].

Along with cross-sectional and longitudinal studies, there are research studies that have tested what happens when people stop using social media, but they are fewer in number. Of the 20 that tested the effects of a digital detox (70% of the 20 specifically restricted social media use), the majority found no effects or mixed findings [10]. The authors concluded that “*mixed findings exist* [i.e., some benefits and some harms of abstaining], but no clear answer can be given yet” [10]. Some social media interventions have studied the change in global well-being after a very short period (eg, 7-20 minutes) [11], which calls into question whether any change could even have occurred in that interval. By comparison, studies that require participants to abstain from social media for a week or more report no changes in daily loneliness, affective well-being, and positive or negative affect [12-14].

One could also ask the question “How much would a person have to reduce their social media use to improve their well-being?” Two studies sought an answer. The first estimated that the change would have to be substantial: “5 hours 8 minutes of daily device-based engagement [would have to occur] before caregivers would be able to notice subjectively significant variations in psychosocial functioning” of their child [15]. Another study of Spanish adolescents estimated that to experience a gain in well-being, social media use would need to be reduced by 10 hours a day [16]. Both estimates far exceed the amount of time typical users spend on social media. Among adolescents and young adults, objectively measured social media time ranges between 2 and 2.5 hours a day [17,18]. For adults, objectively measured daily social media use is only 30-45 minutes [5,19].

Maybe social media use causes stress. When stress is measured through biological methods (eg, cortisol in a saliva sample), studies have found that social media use decreases stress [20,21] or has no association with stress [22]. Substantial reductions in screen time, including time on social media, have no effect on biologically measured stress [23]. One study [24] compared the argument that social media cause stress with the argument that social media are sought out after being stressed. It concluded that stress seems to precede social media use rather than being caused by it [24].

Overall, research suggests that screen time, including social media use, plays little to no role in the well-being of most users. Its effects are not conclusively harmful or toxic in terms of decreasing well-being, increasing ill-being, or causing stress. Rather, the effects of social media on users are negligible but heterogeneous (ie, the experience varies between individuals) [25]. These 2 claims are not inconsistent; it can both be the case that social media do not influence the well-being of most users, but might help or hurt some users in some circumstances.

- *Myth two: Social media addiction is pervasive and harmful.*
- *Warranted claim two: Experts disagree on whether social media addiction exists, what the diagnostic criteria are, and how it should be measured.*

Most Americans (56.9%) believe they are addicted to their smartphones [26], but many experts would disagree with that self-diagnosis. For over 20 years, there has been research on *internet* addiction, which has since accommodated new technologies (ie, the smartphone and social media). However, the *Diagnostic and Statistical Manual of Mental Disorders, fifth edition* (*DSM-5*) does not list internet addiction [27] as a diagnosis and it has not since included social media addiction. Part of the challenge lies in good measurement.

The measurement of technology-based addiction is incredibly fraught. These measures are deeply problematic and major researchers have discounted their value [4,6,28]. The most concerning issue is the tautological relationship between cause and consequence [5,28-30]. Essentially, a person cannot report having a technology addiction without simultaneously reporting a mental health problem [28-30]. One comprehensive review [30] concluded that the measures of technology-based addiction have invalid and arbitrarily designed instruments and major inconsistencies between instruments in terms of conceptual definition, assessment items, and diagnostic criteria. These measurement problems also influence the interpretation of meta-analyses. Specifically, associations between ill-being and internet or social media addiction are substantially stronger than associations between ill-being and time on social media [4,6,31]. Thus, social media addiction research is misleading due to the comorbidity of cause and consequence and poor overall measurement.

The Digital Wellness Lab associated with Harvard Medical School recommends using the phrase “problematic interactive media use” while cautioning that it is not clear whether media use is a cause of an underlying mental health challenge or a manifestation of it [32]. In other words, a large amount of time spent using the internet, smartphones, social media, or video games is probably externalization of psychological disorders, but is unlikely to be the root cause of such disorders [30].

Although a shift from “addiction” to “problematic use” is important, the existing measures of problematic social media use also need further development. Problematic use measures have similar problems as addiction measures, namely that high social media use and mental health impairment are measured using the same items [5,29]. Some researchers have called for a complete reconceptualization of problematic social media use [4,29,30]. Until a valid and reliable measure of problematic use emerges, studies using those instruments should be interpreted with the above limitations in mind.

The myth of social media addiction has major policy implications. Calling something an addiction means it should be *treated* as an addiction. One clear example of how the language of addiction frames public deliberation is the comparison between tobacco use (and tobacco companies) and social media use (and technology companies). This framing justifies the proposed solutions. The argument goes that if social media use is as harmful and addictive as tobacco, then policy makers should combat it as a public health crisis. Indeed, it is common for policy makers and the public to use the drug metaphor to explain how social media function and what the government should do about it [33]. Yet, experts agree that social media addiction is not a diagnosable addiction (ie, it lacks the necessary characteristics of addiction) [27], and, even if it were an addiction, there are no valid measures to document its prevalence. This does not dismiss the possibility that problematic use exists. Rather, to study problematic use, we need better conceptualization and measurement [30] to guide policy decisions and treatment for individuals [32].

- *Myth three: Spending more time on social media will inevitably make users depressed, anxious, sad, and lonely.*
- *Warranted claim three: Over time, declines in well-being are associated with increased social media use.*

The third myth is centered on the question, “Which comes first?” There are 4 possible sequential orders of the association between social media use and well-being over time: changes in social media use decrease/increase future well-being exclusively; changes in well-being increase/decrease future social media use exclusively; both changes affect each other; change in one is not predictive of change in the other. The myth is that social media use *increases* ill-being (or *decreases* well-being) in the future.

The empirical evidence supports the sequential order opposite to this myth: declines in well-being precede increased future social media use. A meta-analysis of longitudinal studies on social media and well-being found that changes in well-being (eg, eudaemonic well-being, hedonic well-being, and social well-being) are associated with changes in social media use, but not the other way around [4]. A longitudinal analysis of 84,000 UK adults aged 10-80 years from 2011 to 2018 found that drops in well-being were followed by more social media use over time across all age groups [34]. The same study, however, found that increases in social media were associated with future declines in well-being for a few adolescent age groups. Other research has found that increased depression predicts future social media use, particularly for adolescents, but not vice versa [35]. Among adolescents, social media use tended to increase after reductions in friendship quality, suggesting that interpersonal struggles precipitate future social media use [36]. Another study found that both declining life satisfaction and higher psychological distress corresponded with increased future social media use among adults [37].

It is important to note that other studies have found no relationship between well-being and social media use over time. One study, including thousands of Dutch adults over 6 years (2012-2017), found no association in either direction [38]. Another identified a bidirectional relationship, but concluded that these associations were so small as to be trivial [39]. Yet another suggested that loneliness and social media use are unrelated over time for adolescents [36]. Sensitive to the difference between well-being and ill-being, a meta-analysis reported that changes in ill-being (ie, anxiety or depression) were unrelated to social media use over time [4].

Another way to frame the discussion is not to focus on change over the years, but to consider shorter time frames, such as within or between days. There are several reasons why people might turn to social media when feeling down, lonesome, or anxious: it is always available, it is distracting, and it could provide a way to get in touch with responsive others [40,41]. But, social media may not be effective at getting one’s needs met. Adolescents commonly use technology to cope with negative emotions, but social media use does not appear to contribute to effective emotional regulation between days [42]. Similarly, people who tend to rely on social media to cope with loneliness do not seem to get their needs met over days or months [43,44]. Thus, people may turn to social media when stressed or down, but social media use does not appear to be effective at helping individuals cope with those emotions.

Focusing on different ways of using social media, a 3-year longitudinal study of late adolescents in Spain examined the use of social media as an escape from problems. Escapism was associated with more time on social media and future declines in well-being [16]. These 3 concepts (ie, escapism, time on social media, and negative outcomes) showed a circular relationship with one another. Perhaps social media functions like a social snack—temporarily redirecting or distracting an individual from their negative affect or loneliness, but failing to fully satisfy their needs or cope with the origin of their negative affect [40,45,46]. For example, during the pandemic, turning to social media to cope with loneliness increased loneliness over time, accounting for the frequency of face-to-face and video chat conversations [47]. Unfortunately, very few studies have focused on how variation in well-being works in tandem with using social media to cope with negative feelings or to escape one’s problems [43]. Thus, more research is needed to determine if some platforms or specific patterns of use are effective for coping or getting one’s needs met.

Although evidence suggests social media use is a poor coping strategy, this is not equivalent to saying that it is directly harmful. Rather, many researchers have suggested that social media use, especially to escape or distract an individual from their problems, is a misdirected solution [4,16,24,42,43,45-50]. This is particularly concerning for adolescents who are already at high risk for ill-being; building a strong habit of social media use to cope may contribute to poor self-regulation [48-50]. Furthermore, because there is some evidence of a bidirectional relationship over time among some adolescents [34], it is possible that using social media to cope could exacerbate existing social, emotional, or mental health challenges. By contrast, because several large longitudinal studies have found nonexistent or trivial bidirectional relationships between social media use and well-being, it is important that future research studies test the “exacerbation explanation” further.

- *Myth four: Social media are the main cause of the problems teens are facing.*
- *Warranted claim four: Preexisting vulnerabilities (eg, poverty, mental health, lack of family support) are associated with both adolescent social media use and adolescent ill-being.*

Some would argue that social media are a primary cause of ill-being because adolescents who are lonely or depressed use social media frequently. When 2 things co-occur frequently, social scientists try to explain why this happens. In pursuit of this goal, researchers have begun to identify what sorts of adolescents are heavy social media users and experience high ill-being. In other words, social scientists look for a third variable that explains both things. If the heaviest social media users also experience high degrees of psychological dysfunction, but *for* *some other reason than social media*, then it could explain the small, but significant, association between social media and ill-being [28,31].

Various preexisting socioeconomic and psychological vulnerabilities (eg, poverty, poor mental health, or lack of family support) precede or co-occur with social media use. Families who are struggling to get by financially and experience frequent stress are more likely to have children with heavier device engagement [13]. A longitudinal study of over 12,000 UK adolescents found that lower family support was associated with greater social media use across time [50]. In the same study, social media use itself did not predict changes in ill-being or well-being [50]. Compared with social media use, family struggles were much more strongly associated with ill-being. An 8-year longitudinal investigation found that when an adolescent was in a riskier home environment (eg, low parental involvement) or was at risk by disposition (eg, low behavior self-regulation), then social media use was related to greater depression over time [51]. Similarly, in lower education and income families, social media use is more prevalent than other types of media or internet use [52]. Adolescents who report problematic smartphone use are more likely to experience stress, poorer health, and less belonging at school [52]. The same study concluded that economically disadvantaged youth report more spillover from online experiences to serious offline problems, including face-to-face confrontations, physical fights, and getting into trouble at school.

By contrast, social media use has little effect on well-resourced users—it may even enhance their well-being. Social media use is associated with greater well-being when people are already socially well-connected [53]. Similarly, adolescents with high well-being are less influenced by long durations of smartphone use [18].

These findings are bolstered by research that separates between-person effects from within-person effects. Between-person effects occur when certain types of people engage in specific media practices. For example, people who are unemployed and socially isolated tend to watch a lot of TV [54]. By comparison, within-person effects occur when media use explains variance in well-being beyond what could be attributable to the between-person effects. Within-person effects in the prior example would mean no matter who the person is, frequent TV watching would be associated with social isolation. When within-person effects are found for media, it rules out the argument that the characteristics of people are the whole story. Research documenting within-person effects has found no association between well-being and social media use [38,55-58]. These studies were conducted across countries, various time frames (days to decades), using large samples, and with longitudinal designs. One meta-analysis of social media use and depression among adolescents confirmed the presence of between-person effects, but not within-person effects [59].

By comparison, there is strong evidence that the association between social media use and well-being lies at the between-person level. This means different types of people engage in different social media practices, rather than social media use *causing* harm. One way to interpret this is to consider the people who have the available time and motivation to be on social media for extensive periods. An abundance of time and a lack of desirable options for leisure or connection may reflect preexisting psychological dysfunction as well as social media users’ socioeconomic or socioemotional context.

Therefore, knowing a person’s background provides a considerable amount of information about their media habits. Whether coming from a “promote the positive” or “mitigate the negative” perspective, researchers have concluded that more attention should be paid to *who* uses social media and *how* they use it. From a digital thriving perspective, researchers have embraced the importance of context in understanding social media use: “We do not interact with our devices in a vacuum, however: The interactive and dialogical nature of digital media implies that our use of them cannot be considered in separation from our social context.” [60]. Researchers operating from a harm mitigation perspective argue that “the conversation surrounding the legislation of social media should focus on determining which segments of the population would benefit from external regulation of social media platforms (e.g., those with psychologically vulnerable dispositions) instead of focusing overtly on the unrealistic end-goal of benefiting all segments of the population by implementing blanket policies.” [58].

- *Myth five: Compared with other harms, the harm of social media use is far greater.*
- *Warranted claim five: Once the primary predictors of well- and ill-being are accounted for, social media use is a negligible factor in explaining variance in well- and ill-being.*

According to popular opinion, it is social media’s fault that bad things are getting worse. For this to be true, among all the factors that cause harm to a person, social media use would have to be a big one. While the fourth myth-claim pair explores the co-occurrence of social media use and a person’s family, socioeconomic, and psychological struggles, the fifth pair explores how important social media use is in the big picture of a person’s overall ill- and well-being.

To proceed, a little background is needed. Early research on social media typically measured social media use (usually the amount of time spent) and then measured global ill-being (eg, loneliness and anxiety) [5], with measures of eudaemonic well-being being relatively uncommon [61]. In these studies, social media use was not measured side-by-side with known indicators of ill- and well-being. Study design influences what we can learn and know. Earlier study designs answered the question “Is social media use associated with ill-being?” More rarely used study designs answer the question “Is social media a stronger or weaker indicator of well- and ill-being compared with other factors?” Combined with a reliance on cross-sectional data, early research on social media could not establish the comparative or relative effect of social media—there was not enough information. Only recently have studies begun to measure social media use side-by-side with other indicators of well- and ill-being.

Social media use explains very little variance in well- and ill-being, compared with other factors. A large study that compared the association between social media use and well-being along with other characteristics of adolescents concluded that “the association of well-being with regularly eating potatoes was nearly as negative as the association with technology use, and wearing glasses was more negatively associated with well-being.” [6]. A large longitudinal study of adolescents concluded that social media are among the least important factors in predicting changes in mental health [50]. A weighted national sample of over 20,000 Americans found that social media use was one of the least valuable predictors of loneliness—and, in this study, more Facebook use was associated with less loneliness [62].

What about social media use compared against other media (eg, TV or the internet)? Studies that sought to answer this question suggest that no form of media use is any more or less predictive of well-being than any other [55-57]. Simply, no form of media consumption seems to matter that much in the big picture. One group of authors concluded “These findings are incompatible with the dose-response model that underlies societal discourse” of social media harms [56]. In other words, once considered in the context of other factors, the equation *X amount of social media use leads to Y amount of harm* does not hold up.

What about over time—does media play an important role there? Long-term longitudinal studies (eg, over several years) are well-equipped to explore gradual changes in ill- and well-being. Such studies suggest that social media use does not matter much [6,7,50,57]. Thus, it is very unlikely that social media use plays much of a role in changes in eudaemonic well-being, life satisfaction, or mental health, which are typically stable and change only gradually [63].

Overall, social media use is a tiny (perhaps nonexistent) factor in predicting ill- and well-being. Other major factors—and even some smaller factors such as wearing glasses—play a bigger role than social media use in understanding whether a person experiences greater well-being or less ill-being. Global well- and ill-being change over long periods or because of major changes in life circumstances; they are probably not meaningfully affected by a person’s preference of media.

- *Myth six: The adoption of social media, especially on mobile devices, perfectly coincides with the beginning of the contemporary adolescent mental health and loneliness crises.*
- *Warranted claim six: Longitudinal studies do not support the conclusion that the adoption of mobile or social media preceded or caused declines in adolescent mental health or the adolescent loneliness epidemic.*

Time spent socializing has been on the decline for at least 30 years in several countries across the global north [54,64-66]. Although social time has declined across demographic groups, unmarried, unemployed adult men appear to have experienced the greatest increase in time alone in the United States in the past 50 years [54,64]. Loneliness is typically high among late adolescents and young adults because it is associated with the developmental challenges of emerging adulthood. However, there has been a meaningful rise in loneliness among adolescents and young adults in the global north [64,66]. In response to these trends, it has been argued that there is a direct relationship between the adoption of social media and smartphones (~2011-2014) and subsequent increases in ill-being. Some people have concluded that social and mobile media have directly caused the rise in loneliness and decline in mental health among the youth.

Longitudinal findings do not support this myth. One study of US adolescents from 2009 to 2017 found that changes in depression were not related to prior social media use or between-year changes in social media use [67]. Rather, this study found between-level associations between social media use and mental health that are consistent with those discussed above (see myth five). A study of German adolescents conducted over the decade in question found no evidence that social media use influenced future depression [68]. An 8-wave longitudinal study (2009-2016) of thousands of adolescents in the United Kingdom measured social media use during the school day and found that between-year associations between social media use and well-being were trivial [39]. A meta-analysis of studies from 2006 to 2018 found that the size of the association between social media use and well-being decreased over those 12 years [4]. In other words, social media use had a weaker association with well-being as studies were conducted closer to the present day. The same study found no drop-off in well-being after 2012.

By contrast, a preregistered meta-analysis of loneliness over 50 years found that loneliness has indeed increased for young adults (mean age of 21 years), but its rise preceded the introduction of social media and the adoption of smartphones by nearly a decade [69]. This means loneliness was increasing and time spent socializing was decreasing well before the widespread adoption of social and mobile media.

Another way to challenge this myth is on a country-by-country basis. One study [70], using data that spanned from 2008 to 2019, examined the portion of active Facebook users in 72 countries (as a percent of 13- to 34-year-olds in the population) in relation to well-being (ie, life satisfaction and negative and positive psychological experiences). Facebook usage data were provided by Meta Platforms, Inc. and the Gallup World Poll provided the measures of well-being. Including data from nearly 1 million people, there was no evidence that rates of adoption of Facebook were associated with changes in well-being for young people. In fact, this study found that an increase in positive experiences was associated with greater Facebook adoption within countries. The same study further demonstrated that Facebook adoption was unrelated to changes in mental ill-being (ie, anxiety, depression, or self-harm) within countries [70]. This study is particularly notable as it used objective measures of social media adoption, which is rare in social media and well-being research [28,63]. Similar to the above cross-country results, 2 other longitudinal studies of adult social media users found that greater social and mobile media use was associated with increases in well-being in subsequent years [71,72].

It is reasonable to argue that declining well-being and rising loneliness may influence social media use (see myth three). It is consistent with the evidence that individuals who find themselves in less social circumstances or who seek to cope with isolation or loneliness may turn to social media to distract, escape, or connect [18,43,44,47]. However, these 3 purposes yield very different outcomes. The use of social media to reach out and communicate with loved ones is most likely to help ameliorate loneliness [18,40,41]. By contrast, social media use to escape or cope with negative emotions may not be effective [18,42-44,46]. Social media does not have to be the cause of declining mental health or increasing loneliness to be an unsuitable coping mechanism for individuals who are already depressed or lonely.

- *Myth seven: Social media are the reason people don’t spend time together.*
- *Warranted claim seven: Social media use does not cause people to stop people from talking to each other face-to-face, rather they are used to help people keep in touch when face-to-face interactions decrease.*

This myth has a name when studied by academic researchers: the social displacement hypothesis. This hypothesis states that rising rates of technology use cause declining rates of face-to-face communication, subsequently reducing well-being. Over the last 20 years, there have been rising rates of social media use and declining amounts of social time across the global north [64,65]. Where does social media time come from? If borrowed from face-to-face social time, it is reasonable to argue that this could reduce well-being. If borrowed from other media, it is unlikely to affect well-being [55,56].

Social media time is probably displacing time spent on the internet and watching TV, just as internet use once borrowed time from TV and other media [73-75]. Patterns of media use by US adolescents between 2006 and 2016 suggest that social media use climbed while all other forms of media use fell (eg, books, magazines, newsprint, or movies at the theatre) [74]. More recently, social media platforms have been used to access broadcast, streaming, and influencer or creator content. From 2018 to 2023, about 45 minutes of entertainment content shifted from being watched on a TV to being watched on a mobile device, often through social media platforms [5,76]. Thus, a substantial portion of mobile screen time is spent doing what might be called “watching TV” on a personal device. These days much of this TV-like content is distributed through social media platforms (eg, TikTok, YouTube).

The social displacement of face-to-face interactions is rarely tested using experimental designs (eg, randomly assigning participants to increase social media use). One study tested displacement by reporting what activities people reintroduce when they abstain from social media for weeks at a time [73]. When abstaining, people mainly spent more time browsing the internet, working, and engaging in household activities [73]. Another study found that after 2-4 weeks of Facebook abstinence, participants reported watching more TV and using the internet more during their time away [73]. Two studies found that when abstaining from a platform, people simply redirect their communication and entertainment to other social media platforms [12,73]. In fact, 3 studies found evidence contrary to social displacement: on days that participants abstained from social media, they engaged in fewer social interactions with weak ties [73,77] and engaged in fewer phone calls, texts, and emails [78].

Rather than displacing social interaction, online communication mirrors offline communication. Social media are used to communicate by already social people [79-81]. A longitudinal study of Dutch adults found that face-to-face communication is associated with social media use, and, over time, increased social media use does not decrease time talking to close others [81]. Another German study found that active social media use is positively associated with face-to-face communication 6 months later [82]. A longitudinal study conducted in the early years of social media adoption (2009-2011) found that social media use was not associated with reduced frequency of face-to-face contact within years or in the future [71]. An 8-year longitudinal study of Norwegian children and adolescents (10-18 years old) found that social media use predicted more time with friends offline in the future [83]. The same study revealed that using social media did not affect the development of social skills (ie, communicating online did not harm the development of social skills in general) [83]. These findings are inconsistent with the social displacement hypothesis.

Social media use, particularly texting and group chats, may compensate for a lack of opportunities to engage in face-to-face interaction, such as long-distance friends and family. On days with fewer face-to-face interactions, people turn to smartphones to communicate [84]. When opportunities for in-person contact decline, people use social and mobile media to connect [41].

- *Myth eight: Teens using their smartphones around each other is a sign of a disconnected and discontented generation.*
- *Warranted claim eight: The effects of co-present mobile use are highly situational and influenced by social norms.*

Using one’s mobile phone in the presence of other people is called co-present mobile use or “phubbing” (phone + snubbing). Co-present device use is not social media use per se, as mobile devices are used for a variety of reasons, increasingly for streaming services, watching sports, and online videos [5,19]. However, a great deal of mobile phone use is social media use as well [5,19,76]. Research on phubbing often uses experimental methods to study how people feel during co-present device use, particularly during first conversations between strangers. A meta-analysis confirmed that co-present device use has several negative social consequences during first interactions, leading to the interaction partner having a negative impression of the phubber [85]. There is longitudinal evidence that chronic co-present mobile use affects adults’ romantic relationships. Individuals who perceive their partners to be overly attentive to their smartphones tend to have fewer positive interactions and more negative moods [86,87].

There is evidence that social media use may diminish well-being when potential conversational partners are nearby. If young adults use social media in the company of close friends and family, they experience lower well-being than when they use social media when by themselves [58]. Perhaps, using social media in good company is an opportunity cost [58,81,88]. Yet, it is also possible that people become more attentive to their devices when they are in socially uncomfortable circumstances [40]. However, there is some evidence that challenges the presumptive harms of co-occurring face-to-face and social media use. One study found that engaging in both within the same period is associated with higher affective well-being among adolescents, compared with face-to-face communication alone [89]. In other words, when adolescents reported that they had both face-to-face and virtual social interactions, they reported the highest momentary well-being.

There are other important contextual factors to consider. One observational study suggested that co-present mobile device use is quite common in real life and has negligible effects on relationships [90]. Using a mobile device near a friend while waiting for a class to start does not lead to attentional conflict for either person and it has no association with relational intimacy. Most of the time, the people waiting together do not even notice that a device has been used. Furthermore, it is quite common for people who enjoy each other’s company to share the content of their mobile devices with one another [91,92]. In such circumstances, co-present device use is a way to share something funny or interesting. After all, watching media together—whether a movie or a meme—can be an interpersonal experience.

Overall, co-present device use may negatively influence relationships, first impressions, conversation quality, and, perhaps, momentary well-being. The negative effects are most apparent when people are in a location where it is not appropriate to use a mobile device, people have high-quality relationship partners to talk to [58], and when people use their mobile device to the exclusion of their conversation partner(s) without offering an explanation or apology [40]. As a consequence, the harms or benefits of phubbing are best understood in the context of interpersonal conversational norms.

- *Myth nine: The solution is to quit or ban social media.*
- *Warranted claim nine: The benefits of social media abstinence vary by person and by patterns of use.*

Social or mobile media detox, reduction, abstinence, or prohibition are frequently offered as solutions to their presumptive harms. Logically, if something is bad for you, then quitting it is a good solution. Recommendations vary from school day bans to dinner table or bedtime bans, to uninstalling all social media apps. Some bans and reductions of social media use are self-driven and consistent with personal goals. To the degree that an individual wishes to use social media less, researchers and practitioners should help them achieve their goals. After all, successful media management promotes personal autonomy [60]. Other bans, however, offer no exceptions for personal patterns of use as they are imposed by governments, schools, or other institutions. The effects of social media prohibition by institutions (eg, a school-wide ban) on their members are understudied and remain a crucial topic for future research. However, there is good reason to believe that social media abstinence is unlikely to achieve the desired aims for all users equally.

One reason for the lack of efficacy is that there are benefits associated with social media use. The use of social network sites, particularly Facebook, is associated with online social capital [93]. When compared with online games and general internet use, social media use is particularly beneficial for cultivating bridging social capital, which provides access to unique information and opportunities [3]. Through social network sites, users have access to diverse sources and can ask for or lookup information or aid. A recent research review confirmed the positive association between social media use and online social capital across the world, including the global south (eg, Pakistan, Iran, Palestine, Bangladesh, and China) [94]. When social media enable access to online communities, abstinence from social media could be a loss of access to information, aid, and support. Furthermore, abstinence would be a loss for people who have offline communities that expect group members to be available on social media, including friendship groups, workplaces, teams, or volunteer organizations. Leaving social media means not being informed about one’s offline community too [40,60,77]. It is important to note that this online availability may create its own pressure to be constantly responsive and available, which may interfere with users’ autonomy and well-being by creating digital stress [17,40,60].

A second benefit is associated with engaging in one-on-one or one-to-few conversations, such as texting or group chats through social media (eg, Snapchat, WhatsApp, or Facebook Messenger). Texting, sharing content, and responding to others’ content (sometimes called *active* social media use) may be weakly associated with global, eudaemonic, and social well-being [4,28]. A recent meta-analysis suggested that the difference in effect between passive and active social media use is probably negligible, however, partly because both effects are very small [95]. It may depend on whom a social media user is communicating with [40]. Among Spanish young adults, using social media to communicate with friends and family is associated with greater well-being over time [16]. Chatting online and giving feedback to others are both associated with positive emotions and affective well-being for Dutch and German adolescents [25,96]. Indeed, texting frequency is associated with global well-being and reduced loneliness [9,43] and is particularly important for maintaining long-distance romantic relationships and friendships [97-100]. For users of social media in supportive social environments, texting is associated with reduced depression in longitudinal research [101]. In fact, a moderate amount of texting has been shown to offer a protective effect for some adolescents, even for those in socially disadvantaged environments [101]. This suggests blanket bans on social media use, especially ones that prevent mobile texting and social media direct messaging, will not help those who are most vulnerable.

The third benefit of social media use is in the domain of hedonic well-being. Social media content is often enjoyable, funny, and inspiring [102]. Italian adolescents have found that smartphone use is associated with short-lived hedonic pleasure [18]. Social media use may influence eudaemonic well-being, particularly when users seek content that is inspiring, motivational, and growth oriented, such as learning new skills [60,102].

The consequences of quitting social media can be found in studies that experimentally assign people to reduce use or abstain altogether. In interventions for social media use that require abstinence, 26% of 39 studies reported declines in well-being after leaving social media, particularly in the domain of social connection [11]. Meta-analyses suggest that social media use is more strongly associated with social well-being than it is negatively associated with anxiety or depression. These associations are drawn from reviews that include social media addiction studies, which tend to inflate negative effect sizes [4].

An exemplary summary [103] of when and why abstaining from social media would be helpful concluded that the benefits of quitting are not straightforward—even for people who want to. People feel guilty about using social media because they believe that it is harmful or unproductive, or both [103,104]. Part of the negative feelings associated with mindlessly scrolling is due to the guilt about doing so [105]. Therefore, successfully abstaining from social media can bring about a boost of well-being (“I stopped something that is bad for me” or “I accomplished my goals”) [13]. But, failure to break a strong habit can lead to negative self-evaluations (“I failed to achieve my goal”) [103]. This could be particularly pernicious if self-control failure is how individuals arrived at the conclusion that they need to digitally detox in the first place. For example, mindless scrolling is most strongly associated with goal conflict for those with the least self-control [105]. It is likely that self-control failure both contributes to frustrations with social media and makes it difficult to break well-established media habits. When people try to restrict their use by changing their notifications or smartphone-specific reminders, they experience more anxiety, worry, and check their mobile devices even more [103]. This suggests that technological solutions may not provide a quick fix—quitting an ingrained habit is difficult and aversive.

Finally, it is important to point out that restricting mobile communication may be particularly harmful to those who need access to their online community the most. Approaches developed from the need to belong framework have suggested that mediated communication with loved ones is better than no communication, especially when people are going through times of social deprivation [40,41,99]. People who increased mediated communication at the beginning of the pandemic reported significantly more closeness to friends and family compared with those who did not [47]. As an important cautionary note, this benefit does not seem to apply to all types of social media use [43,46,57]. Whether texting and active social media use are sufficient to meet individuals’ fundamental need to belong or to ameliorate loneliness over longer periods (eg, weeks, months, or years) requires further study.

- *Myth ten: We do not need another study on social media.*
- *Warranted claim ten: The research on the harms or benefits of social media must continue as platforms, features, habits, contexts, and users constantly change.*

Some prominent researchers and advocates speak definitively and without reservation about the harms of social media. They often find an audience who agree with their warnings. After all, belief in the harms of social media is widespread. Other researchers offer nuanced, hedged, and complex responses to the question of harm [103,106,107]. Embracing the ideas espoused in this paper—for example, that social media are a small player in a big stage of adolescent development and do not have a causal effect on well-being—will be unsatisfying to many people. It may fly in the face of many adolescents’ and parents’ personal experiences or beliefs. Clear certainty of an obvious villain is intuitively more satisfying than answers that resemble “it’s complicated” and “we need more research.” It is completely understandable if the public is frustrated with the state of social media research (or the contents of this paper).

Researchers themselves disagree about how to interpret the results. Where some see substantial or clearly detrimental results, others see trivial or meaningless effect sizes [6,28]. Two well-designed studies of the same phenomenon will find different results. This is due to the nature of social science; each study contributes a little more information to an incomplete puzzle. Furthermore, social media are always changing, which means the puzzle picture itself is in flux as researchers seek to assemble it. By the nature of science and by the nature of the topic, research on social media will never be definitive, unchanging, and 100% accurate. It will always be probable and contingent. And researchers *should* always be open to being wrong. Good research is open to criticism and improvement and recognizes contingencies and nuance. The warranted claims offered in this paper may prove wrong as more evidence mounts. As unsatisfying as it may be to policy makers and the public, this is how good science works. In the meantime, we need more research, with better designs, and better theory to explain when and why social media might affect well-being.

Vanden Abeele and her colleagues [33] offered an intuitive way to think about social media. They present 3 metaphors about its nature: a drug (ie, the addiction framework), a demon (ie, insidious platform design), or a donut. They argue that the final metaphor is most useful and compelling. A donut may not be the best source of nutrition, but whether it is bad for a person depends on the characteristics of the person and the context. If it is wanted and not a substitute for nutritious food, then it is probably fine. On any given day, eating a donut is not going to change the trajectory of a person’s life. Eating donuts, no matter how delicious, will not make a person happy or healthy in the long run. Eating a lot of donuts is a bad coping mechanism for real problems. A habit of excessive donut eating in response to real interpersonal or mental health struggles will create new problems. But this does not mean that donuts caused the initial unhappiness.

The donut metaphor is also useful because it is just one food on a menu of options that vary in healthfulness. Social media use is just one way of occupying time from a menu of media options that occupy a lot of people’s leisure time [54]. Even if we believe that social media are harmful, is it any more harmful than TV as a source of amusement or distraction? Probably not [55-57]. This comparison is especially relevant as TikTok and YouTube become more popular and as more people stream more TV content through social media.

The benefits or harms of social media depend on what a person needs, the choices a person has, what their media diet is, and the degree to which their habit is chronic and excessive [33,60]*.* To be clear, social media use is probably more harmful to some people than others [6,103], such as those with poor executive functioning, those with poor social and emotional resources, and those for whom social media are the only good option for finding connection and meaning [52,58,60]. Personality does not seem to be a source of this moderation, but global well-being matters quite a bit [57,58]. For example, a large study of social media use among late adolescents found that individuals with higher depression and lower life satisfaction tended to have a more negative experience with social media [58]. Lonely people experience more loneliness on days with more social media use, while well-connected people tend to feel more connected on days of more social media use [53]. Beyond these preexisting vulnerabilities, social media are a considerable source of distraction for most adolescents, particularly among those with low executive functioning [108].

The donut metaphor directs future research to develop a better understanding of *when* and *for whom* social media have negative effects. It also cautions against researchers (or the public) assuming that a donut is not food for someone who is hungry, or social media are not a source of amusement and connection for someone who is alone. Conversations about hyper-palatable foods, such as donuts, are instructive. We may be quick to criticize people who eat chips and processed foods or drink soda, but we must keep in mind that those foods may be the cheapest and most accessible form of nutrition a person has (eg, they are living in food deserts). Although it is easy, and may feel morally righteous, to tell a person who is depressed or lonely that they should not use social media to cope, we must consider what other forms of social nutrition that person has available to them. This perspective suggests that research should study ways to help individuals—as well as parents and teachers—build better coping mechanisms and ways to manage their media habits [109]. Blaming social media distracts policy makers and stakeholders from focusing on the root causes of loneliness, mental health struggles, and a lack of life satisfaction or meaning [1].

Finally, the donut metaphor applies to the benefits of social media as well as its harms. It makes sense that donuts made from higher-quality content are probably healthier (and more delicious). Social media content changes depending on whom someone is communicating with or watching. However, researchers do not know if the benefits of using social media are due to predisposition or existing social resources rather than content. Are there specific patterns of social media use that would be beneficial for everyone, or are positive social media habits nontransferrable between people? Although intuitive, encouraging more active and less passive use does not seem to be a solution [95]. Establishing the nature of beneficial social media use might provide clarity about how to formulate interventions and promote healthy mobile and social media habits.

In the bigger picture, we must accept that social media use is something that humans created and continually recreate. The collapse of once-popular social media platforms is a good reminder that no platform is permanent. For the user, social media habits exist at the intersection of agency, preferences, predisposition, context, and leisure. Rather than approaching social media as something that should be forbidden if it is not beneficial, it is better to consider social media as a choice of how people use their leisure time. For most people, spending time using social media is neither beneficial nor harmful—it is merely a small piece of a bigger whole. Although it is not a great idea to make a compulsive habit out of social media (or donuts) and doing so will not solve preexisting challenges or vulnerabilities, social media do not cause the harms purported in the 10 myths either.

## Abbreviations

DSM-5: *Diagnostic and Statistical Manual of Mental Disorders, fifth edition*
